# Antibiotic in myrrh from *Commiphora molmol* preferentially kills nongrowing bacteria

**DOI:** 10.2144/fsoa-2019-0121

**Published:** 2020-02-20

**Authors:** Mrinal K Bhattacharjee, Tahrir Alenezi

**Affiliations:** 1 Department of Chemistry & Biochemistry, Long Island University, Brooklyn, NY 11201, USA; 2 Department of Biology, Long Island University, Brooklyn, NY 11201, USA

**Keywords:** antibiotics, bactericidal, *Commiphora molmol*, dormant bacteria, myrrh, nongrowing bacteria, persisters

## Abstract

**Aim:**

To demonstrate that myrrh oil preferentially kills nongrowing bacteria and causes no resistance development.

**Method:**

Growth inhibition was determined on regular plates or plates without nutrients, which were later overlaid with soft agar containing nutrients to continue growth. Killing experiments were done in broth and in buffer without nutrients.

**Results:**

Bacterial cells were inhibited preferentially in the absence of nutrients or when growth was halted by a bacteriostatic antibiotic. After five passages in myrrh oil, surviving colonies showed no resistance to the antibiotic.

**Conclusion:**

Myrrh oil has the potential to be a commercially viable antibiotic that kills persister cells and causes no resistance development. This is a rare example of an antibiotic that can preferentially kill nongrowing bacteria.

## Lay abstract

Myrrh resin has been used for centuries in many cultures for its medicinal properties and has also found its place in most religions. However, due to incorrect testing methods, the antibiotic property of myrrh has been found to be very weak. We report here that myrrh has a strong and unique antibiotic activity preferentially against nongrowing bacteria, a property not found in any commercially available antibiotic. This unique property, along with its low toxicity and less tendency of antibiotic resistance development, suggests that myrrh can be developed to be a promising and ideal antibiotic with lower dosage requirements.

Most of the antibiotics in use today were discovered in the first five decades after the discovery of penicillin, the first commercial antibiotic. Very few new antibiotics have been developed in recent times and very few are in the pipeline. Meanwhile, antibiotics, which were once called miracle drugs, are no longer as predictably effective today. The main reason for this is that overuse and inappropriate use of antibiotics result in development of resistant bacteria [1].

Another reason for the ineffectiveness of antibiotics is their inability to kill nongrowing cells. Even nonresistant bacteria can withstand antibiotics for varying times, which is the reason why antibiotic treatments need to be continued for several days. Because of phenotypic variations of the infecting bacteria in host tissues, there are always some bacterial cells that are slow growing or nongrowing and less responsive to antibiotics, thus requiring extended treatment [2]. Some examples of infections by slow growing bacteria are urinary tract infections, tuberculosis and leprosy [3,4]. Discovery of most of the antibiotics in clinical use today involved testing their activities *in vitro* on exponentially growing cells. Thus, it is widely believed that most of these antibiotics are effective only on growing cells. In a classical study by Eng *et al.* [5] it was demonstrated that except for fluoroquinolones and ofloxacin, all antibiotics tested had very little activity on nongrowing cells. It is known that treatment of bacteria with bacteriostatic antibiotics, reduces the efficacy of those bactericidal antibiotics that are effective only on growing cells [6].

Bacteria may be nongrowing for several different reasons: they may enter a stationary phase due to lack of nutrients; in a population of growing cells there may be a genetically identical sub-population of nongrowing cells, which are known as persisters; they may be nongrowing or slowly growing when bound to solid surfaces such as biofilms on prosthetic devices or when inside phagocytes; and their growth may be stopped by treatment with bacteriostatic antibiotics, which inhibit growth of bacteria but do not kill them. In a recent study by McCall *et al.* [7] it was demonstrated that contrary to popular belief that most antibiotics cannot kill nongrowing cells, there are actually numerous antibiotics that are capable of killing both Gram-positive and Gram-negative cells that are not growing irrespective of the reason for their nonreplication. However, even in this expanded list of antibiotics, there are hardly any that can preferentially kill nongrowing compared with growing bacteria. In this study we report that the resin myrrh from the thorny plant, *Commiphora molmol* preferentially kills nongrowing bacteria at a fast rate, thus representing one of the first known examples of antibiotics with such property.

Many of the top-selling drugs are natural products or their derivatives including those obtained from plants [8]. *Commiphora molmol* (aka *Commiphora myrrha*, common name: myrrh) is an aromatic plant belonging to the Burseraceae family, also known as the torchwood or incense family [9]. Two members of the family, frankincense and myrrh, have been used as perfume, incense and medicine dating back to thousands of years in all parts of the world and have found their place in most religious practices. Myrrh is naturally grown in India, East Africa and Saudi Arabia. The reddish gum resin (called myrrh) is the hardened form of the sap that is extracted by making longitudinal cuts in the tree trunk. Myrrh has been used in traditional medicine as a remedy for mouth injuries, colds and improvement of wounds [10]. However, systematic scientific study of its antibiotic activity is very limited. One possible reason for this lack of positive result is the traditional methodology for testing of antibiotic activity, which is always performed on growing bacterial cells. However, we have discovered that this antibiotic has the unique property of preferentially killing nongrowing bacteria. We present here a modified method to demonstrate the strong antibiotic activity of myrrh against nongrowing bacterial cells. This is of great significance since the inability to kill nongrowing cells is one of the main reasons for the ineffectiveness of most antibiotics currently in use.

## Materials & methods

### Isolation of myrrh extract

*Commiphora molmol* (myrrh) resin was purchased from an herbal store in Riyadh, Saudi Arabia. Myrrh resin (2.0 g) was ground to fine pieces using a mortar and pestle and soaked in 5 ml of 95% ethanol three-times and decanted. The combined ethanol extract was centrifuged at 12,000 × *g* for 10 min and the supernatant was collected. The ethanol was evaporated under reduced pressure, which left 0.97 ml (0.762 g) of oil. Since the oil is insoluble in water, a 20% v/v (corresponding to 15.7 % w/v) solution of the oil in ethanol was used as a stock solution for all experiments. All percent concentrations in this report are expressed as w/v.

### Bacterial strains & culture conditions

*Escherichia coli* (MV10) and *Staphylococcus aureus* (ATCC 25923) were grown in Luria Bertani (LB) medium at 37°C. Nutrient-free phosphate plates were prepared by the same method as other plates except that sodium phosphate buffer pH 7.0 was added to a final concentration of 10 mM instead of LB. When needed, various amounts of 15.7% (w/v) myrrh oil stock solution were added prior to pouring on plates.

### Inhibition studies

Because of the insolubility of myrrh oil in water, minimum inhibitory concentration (MIC) experiments could not be done in broth. So all MIC experiments in this study were done on plates. Cells were grown overnight and serial dilutions were spread on plates containing either LB or phosphate buffer and various concentrations of the myrrh oil. LB agar plates were incubated overnight and the colonies that grew were counted. Nutrient-free phosphate plates were first incubated for 1 h, during which time the cells could not grow because the plates contained no nutrients. Growth was resumed for the cells that survived antibiotic treatment by pouring 3.5 ml soft agar (0.6%) containing LB at 45°C on top of the phosphate-agar plates. After incubating the plates for 24 h at 37°C, colonies that grew were counted. For all experiments involving counting of colonies, weighted averages of the different serial dilution plates were calculated. MIC is defined as the concentration of the antibiotic that stopped the growth of >99.9% cells.

Zones of inhibition were determined by Kirby Bauer disk diffusion method followed by background staining for greater visualization [11].

### Rate of killing of cells by myrrh oil

Rate of killing was determined for both growing and nongrowing cells. From an overnight culture of cells, 0.2 ml was centrifuged, the supernatant was discarded and the cells were resuspended in 2.0 ml of nutrient-free 0.02 M phosphate buffer (pH 7.0) and 1 ml each was distributed into two microfuge tubes. To the first tube, 40 μl of ethanol was added as a control. To the second tube, 40 μl of 15.7% myrrh oil was added to a final concentration of 0.60% in the absence of nutrients. The tubes were incubated at 37°C. Serial dilutions were spread at indicated times on LB plates, which were then incubated overnight at 37°C and colonies that grew were counted. To determine the rate of killing of growing cells, the same experiment was performed, except that phosphate buffer was substituted with LB medium.

## Results

### Myrrh oil preferentially inhibits nongrowing cells

**Figure 1.**
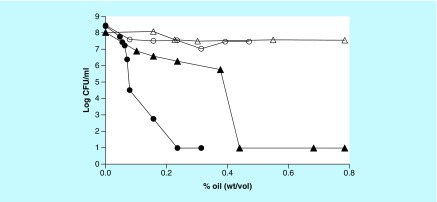
Minimum inhibitory concentration determination on plates. Effect of different concentrations of myrrh oil against *Escherichia coli* (triangles) and *Staphylococcus aureus* (circles) on Luria Bertani plates (open symbols) and phosphate plates (filled symbols). Approximately the same number of cells was spread on plates containing indicated concentrations of myrrh oil. CFU: Colony-forming unit.

Initial attempts to demonstrate antibiotic activity of myrrh oil against *E. coli* on LB plates were disappointing because the extent of inhibition at 0.24% oil was only about 70% and did not increase at higher oil concentrations as shown in Figure 1. However, when the same experiment was done on nutrient-free phosphate plates as described in materials and methods, a dramatic increase in inhibition was observed. At 0.44% (4.4 mg/ml), all *E. coli* cells were prevented from growing. Note that a log colony-forming unit of 1, corresponding to 10 cells/ml, is the minimum limit of detection by this method since 0.1 ml cells were spread. This demonstrates that myrrh oil preferentially inhibits nongrowing cells on phosphate plates but not growing cells on LB plates. The same result was obtained with *S. aureus* for which there was very little inhibition on LB plates but all cells were inhibited from growing at 0.24% oil concentration on phosphate plates (Figure 1). The data also demonstrate that myrrh oil had a much stronger activity on *S. aureus* (MIC 0.079%) than on *E. coli* (MIC 0.44%).

### Myrrh oil contains a bactericidal antibiotic

**Figure 2.**
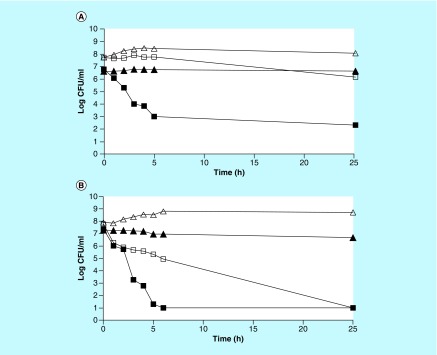
Rate of killing by myrrh oil. CFU surviving after exposure of **(A)***Escherichia coli* and **(B)***Staphylococcus aureus* to 0.60% myrrh oil (squares) or equal volume of ethanol (triangles) in 1 ml Luria Bertani (open symbols) or phosphate buffer (filled symbols). CFU: Colony-forming unit.

The MIC experiment described above does not differentiate between bacteriostatic and bactericidal antibiotics. In order to demonstrate the bactericidal activity of myrrh oil, a rate of killing experiment was done with *E. coli* and *S. aureus* in LB medium, as well as in nutrient-free phosphate buffer. Control tubes contained equal volume of ethanol that is present in the stock solution of the oil. The results in Figure 2 demonstrate that myrrh oil preferentially kills nongrowing cells in buffer compared with growing cells in LB medium. In a nutrient-free buffer, 99.9% cells were killed in approximately 4 h for *E. coli* and in <3 h for *S. aureus*. There were no surviving cells remaining after 6 h for *S. aureus*, whereas for *E. coli* there were >200 surviving cells per ml even after 25 h. Thus, myrrh oil has stronger bactericidal activity on nongrowing cells of *S. aureus* than on *E. coli*. When the experiment was done in LB medium to allow cell growth, *E. coli* showed a slight initial increase in the number of cells due to presence of nutrients. This was followed by a very slow rate of killing of only 1.7 log in 25 h. On the contrary, *S. aureus* cells were killed by myrrh oil even in LB medium, although, at a much slower rate than nongrowing cells in a nutrient-free buffer. In LB medium it took 25 h to achieve the same extent of killing as was obtained in 6 h in a nutrient-free phosphate buffer (Figure 2).

The bactericidal activity of myrrh oil was found to be temperature dependent. All experiments described in this report were performed at 37°C. If *E. coli* cells were exposed to myrrh oil in nutrient-free buffer at 25 or 4°C, there was no significant loss of cell viability even after 24 h (data not shown).

### Increased bactericidal activity of myrrh oil in combination with a bacteriostatic antibiotic

**Figure 3.**
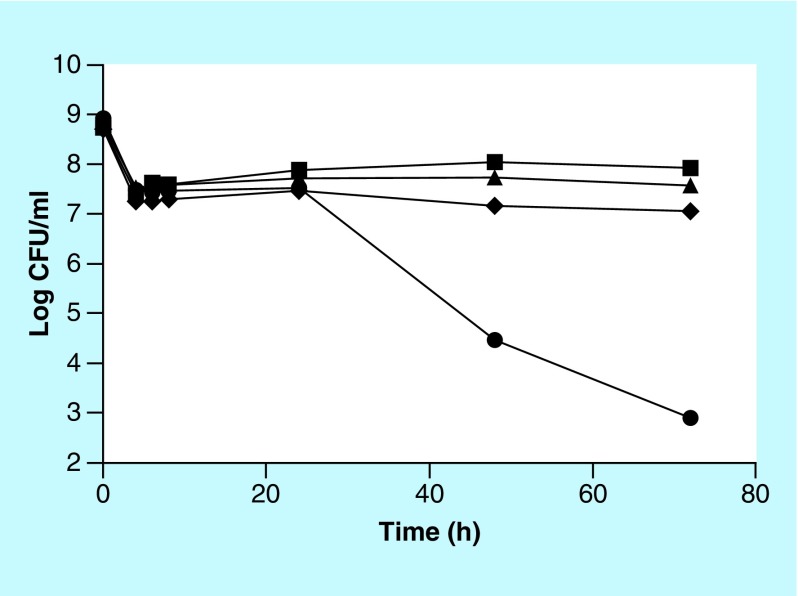
Increased bactericidal activity of myrrh oil in combination with a bacteriostatic antibiotic. Viability of *Escherichia coli* cells after incubation in nutrient-rich (Luria Bertani) medium with the following additions: none (squares), 10 μg/ml chloramphenicol (triangles), 0.60% myrrh oil (diamonds), and 10 μg/ml chloramphenicol + 0.60% myrrh oil (circles). CFU: Colony-forming unit.

Inhibition studies in phosphate buffer demonstrated that myrrh oil has preferential activity on nongrowing cells (Figures 1 & 2). However, since a nutrition-free medium is not clinically relevant, it is desirable to have antibiotic activity even in the presence of nutrients. One possible way to have nongrowing cells even in a nutrition-rich medium is to use a bacteriostatic antibiotic, which can stop the growth of the bacteria. Chloramphenicol was selected as the bacteriostatic antibiotic to be used in combination with myrrh extract. First MIC of chloramphenicol against *E. coli* was determined in broth to be 3.5 μg/ml (data not shown). Antibiotic activity of a combination of the two antibiotics against *E. coli* was determined as follows. Overnight culture of *E. coli* was centrifuged and cells were resuspended in fresh LB plus either 0.60% myrrh oil or 10 μg/ml chloramphenicol or a combination of the two. Cell viability was determined at indicated times as described previously. Since the concentration of chloramphenicol used (10 μg/ml) is much higher than the MIC (3.5 μg/ml), it is expected that the cells will not be growing even in a nutrient-rich LB medium and thus can be killed by the myrrh oil. The results in Figure 3 demonstrate that neither chloramphenicol, nor myrrh oil alone, had any bactericidal effect in LB medium. However, a combination of the two had a strong bactericidal activity on *E. coli* cells. Once again this experiment reconfirms that myrrh oil has antibiotic activity on nongrowing cells only. Therefore, myrrh oil has the potential to be a clinically useful antibiotic especially when used in combination with a bacteriostatic antibiotic, even in a nutrient-rich medium.

### No resistance development against the antibiotic in myrrh oil

Resistance development is a major concern for most antibiotics that are currently commercially available. Bacteria that survive antibiotic treatment are either persisters or have developed genetic mutations, which make them resistant to the antibiotic. *E. coli* cells were tested for their resistance development to myrrh.

First an overnight culture of cells was centrifuged and the cell pellet was resuspended in nutrient-free phosphate buffer. The cells were then exposed to 0.60% myrrh oil in 1 ml nutrient-free phosphate buffer for 24 h and then 100 μl was spread on LB plate. This process was designated as the first passage. One of the colonies that grew was again grown overnight in broth and used to repeat the same experiment for the second passage. This way the experiment was continued for five passages. If the colonies that grow are resistant mutants, then in each successive passage the percentage of cells killed should decrease. The results in Table 1 show that even after five passages all cells were killed by the myrrh oil. Thus, there is no resistance development even after five passages. It is to be noted that no colonies were obtained after spreading 100 μl from the second passage. So the remaining 900 μl cells were used to inoculate a fresh culture, which eventually grew and was used to continue with the remaining passages.

**Table 1.** Surviving cells are persisters and not resistant mutants.

Description	CFU/ml (10^6^)				
Passage number	1	2	3	4	5
Control^†^	18.6	17.0	13.8	8.9	9.5
0.60% myrrh oil	0.00344	<0.00001^‡^	0.00471	0.00004	<0.00001^‡^

† Control tubes had equal volume of ethanol as is present in the myrrh oil extract.

‡ Limit of detection based on 100 μl cells spread on plates.

CFU: Colony-forming unit.

A similar conclusion was arrived at for *S. aureus* by doing zone of inhibition experiment. With 3 mg of the myrrh oil spotted on a disc, a 1.2 cm diameter zone of inhibition was obtained on LB plate (data not shown). Within the zone of inhibition, there were a few small colonies growing. When one of these small colonies was grown in broth and then used as the cells for a second zone of inhibition experiment, a similar size zone was again obtained (data not shown). This suggests that the few colonies growing within a zone of inhibition are persisters and not resistant colonies.

## Discussion

Persister cells are a major cause for the lack of effectiveness of antibiotics. Their resistance is not due to any genetic mutation but is a result of their lack of metabolism. Later, if an antibiotic is removed, these persister cells can continue to grow [12]. In the resting state of the persisters, cell wall synthesis and protein biosynthesis are downregulated and thus these targets cannot be inhibited by antibiotics. Moreover, the cell envelope’s thickness in persister cells often increases, making it difficult for antibiotics to get into the cell [13]. Several methods have been developed to kill persister cells [14]. Examples include using mitomycin C [15] and cisplatin [16], which can enter the cells without the need of active transport and thus can enter persister cells. Another approach to make antibiotics more effective is to wake persister cells by adding sugars [17] or cis-2-decenoic acid [18] so that they can then be killed by traditional antibiotics. A yet another approach reported was to re-engineer tobramycin by adding a 12 amino acid transporter sequence, which allowed it to spontaneously permeate membranes of persister cells [19]. Plant products have been shown to kill persister cells. Essential oils from several spices were found to have activity against the *Borrelia burgdorferi* stationary phase culture [20]. Treating lyme disease, which is caused by the bacteria, is often difficult due to the presence of persister cells of the bacteria.

Killing effects of antibiotics are known to be dramatically reduced in the presence of foreign bodies such as sutures and implants because bacteria form biofilms on the foreign bodies [21]. For example, catheter associated urinary tract infections are of serious concern [22]. Cells in biofilms are slow growing and thus are more resistant to antibiotics. It was shown that *Aggregatibacter actinomycetemcomitans* produces the glycoside hydrolase, dispersin B to degrade its own biofilm [23]. The same enzyme can be potentially used to disrupt *Pseudomonas aeruginosa* biofilms and make the cells more susceptible to antibiotics [24]. Other approaches for making the cells in a biofilm more susceptible to antibiotics are disruption of the biofilm with human Dnase I [25], using diarylquinolines that can kill both planktonic cells as well as those growing in biofilms [26]; and using a combination of the acyldepsipeptide antibiotic, ADEP4 and rifampicin, which completely eradicated *S. aureus* biofilms [27]. Another approach to boost the bactericidal activity of antibiotics against persisters and bacteria in biofilms is by adding exogenous metabolites to stimulate their central metabolic pathways [28]. Strategies against methicillin-resistant *S. aureus* persisters have been reviewed [29].

In this study, our discovery of the action of myrrh resin extract on nongrowing cells opens up a different strategy for combating bacterial infection. Unlike other known antibiotics, myrrh oil will preferentially kill nongrowing cells and in combination with a bacteriostatic antibiotic such as chloramphenicol, all cells in the population could also be killed. Clinical situations in which bacteria can be nongrowing also ensure that the concentration of the bacterial cells will be low. Thus antibiotics that kill only nongrowing cells will have the added advantage that the amount of endotoxins released after their bactericidal activity will also be less. Since myrrh oil and chloramphenicol combination is able to kill both growing and nongrowing cells, it will not be necessary to continue the antibiotic treatment for many days. Thus bacteriostatic antibiotics such as chloramphenicol, whose use was once approved but later discontinued due to minor toxicity, can be reconsidered for use in combination with myrrh.

The results in Table 1 determine that in spite of several passages, no resistant mutant could be obtained because after each passage the cells were equally sensitive to the antibiotic effect of myrrh oil, demonstrating close to 100% cell death. This may have great commercial significance since most of the currently used commercial antibiotics have become less effective due to the development of resistance [28]. It is to be noted that since resistant mutants obtained during growth of any bacteria can only be due to point mutations, the lack of resistant mutants in this experiment does not rule out the possibility that other strains of bacteria in nature may have evolved to form a resistance gene to counteract the effect of myrrh. Such a gene, if it exists, has not yet been discovered.

The target of the antibiotic in myrrh is not known. However, it can be expected that the target will be a biochemical process taking place in dormant or nongrowing cells. One possible site of action could be the bacterial membranes or membrane-associated enzymes. Disrupting the bacterial membrane bilayer or proteins in the membrane of dormant bacteria is a strategy for treating persistent infections [30]. Daptomycin, a lipopeptide antibiotic that targets the cytoplasmic membrane of Gram-positive bacteria, was shown to have bactericidal activity on both growing and nongrowing cells and remained bactericidal against cold-arrested *S. aureus* [31]. The lack of antibiotic activity of myrrh at 25 and 4°C may indicate that the membrane may be frozen at these temperatures and thus not allow transport of the antibiotic into or through the membrane. The facts that myrrh extract is an oil and that no resistant mutants could be obtained (Table 1) are both consistent with the possibility that the site of action of myrrh could be the membrane. A similar observation was made with membrane-targeting AM-0016, which kills mycobacterial persisters and shows low propensity for resistance development [4]. However, these observations cannot explain why the antibiotic in myrrh preferentially inhibits only nongrowing cells. Interestingly, this is similar to a report that *P. aeruginosa* cells in the exponential growth phase were resistant to the membrane acting macrolide antibiotic azithromycin, while cells in the stationary phase were susceptible [32]. Further research is needed to understand the mechanism of action of the antibiotic in myrrh. Today as we are going through an antibiotic crisis, scientists are increasingly looking into plant products for a solution [33]. Myrrh resin can be a promising source of a future antibiotic. Other uses of myrrh have also been reported. For example, it was shown that myrrh and vitamin C synergistically minimize the toxic effects of the macrolide antibiotic, tilmicosin, through their free-radical scavenging and potent antioxidant activities [34]. There is some confusion in the scientific community about whether plant products can be called antibiotics. The original definition of antibiotics as proposed by Selman Waksman >70 years ago, required that they have to be of microbial origin. However, that definition was too restrictive. Sulfa drugs, the first commercially marketed antibiotics that have saved millions of lives were synthetic drugs and not of microbial origin. In fact, the majority of successful antibiotics in use today are either synthetic or semi-synthetic and are actually better than the natural ones due to the reduction in resistance development. It is our opinion that the definition of an antibiotic should not be unnecessarily restrictive, but should rather be more inclusive. It should be based on function and not on the source of the antibiotic [1]. Another drawback of the original definition of antibiotic is that it did not address the concept of selectivity or toxicity. Although we have not tested the toxicity of myrrh oil, it is not expected to have significant toxicity since it has already been in use for centuries. This makes it an even more ideal antibiotic.

## Conclusion

We have demonstrated strong antibiotic activity present in myrrh resin and there is no evidence of resistance development even after repeated passages. It preferentially kills nongrowing bacteria compared with growing bacteria, thus is different from most other antibiotics known and thus has the potential to be a commercially viable antibiotic that can kill persister cells.

## Future perspective

With very few new antibiotics being developed, plant products represent promising sources of antimicrobial agents. Preferential killing of nongrowing bacteria by myrrh oil makes it a promising candidate for development as a future antibiotic. Although toxicity studies have not yet been done, its use for centuries in traditional medicine suggests that its toxicity will be low. Future studies will focus on purification and identification of the active component in myrrh oil, its mechanism of action and pharmacokinetic properties including toxicity.

**Summary points**

- Most antibiotics are unable to kill nongrowing bacteria, which is the reason why antibiotic treatments need to be continued for several days.
- Although there are some antibiotics that have activity against both growing and nongrowing cells, there is almost no antibiotic that is specific for nongrowing bacteria.
- We show here that myrrh oil from *Commiphora molmol* preferentially kills nongrowing cells. Cells are killed much faster in buffer without nutrients than in broth rich in nutrients.
- Possible clinical significance of myrrh oil can be that it can also kill bacteria in nutrient-rich media provided growth of the bacteria is halted by addition of a bacteriostatic antibiotic such as chloramphenicol.
- Another positive aspect of the use of myrrh oil as an antibiotic is that even after repeated use of the antibiotic there is no evidence of resistance development. This property is similar to that of membrane-acting antibiotics.
